# P-1194. Activity of manogepix against mould isolates collected from the US in 2023

**DOI:** 10.1093/ofid/ofaf695.1387

**Published:** 2026-01-11

**Authors:** Marisa Winkler, Samuel Edeker, Abby Klauer, Paul Rhomberg, Mariana Castanheira

**Affiliations:** Element Materials Technology/Jones Microbiology Institute, North Liberty, Iowa; Element Materials Technology/Jones Microbiology Institute, North Liberty, Iowa; JMI Laboratories, North Liberty, Iowa; Element Materials Technology/Jones Microbiology Institute, North Liberty, Iowa; Element, North Liberty, IA

## Abstract

**Background:**

Manogepix (MGX) is a novel agent targeting the fungal Gwt1 enzyme. This agent is undergoing phase 3 clinical trials for invasive candidiasis and mould infections. Here, we assessed the *in vitro* activity of MGX against US mould isolates from invasive infections.

**Figure:**
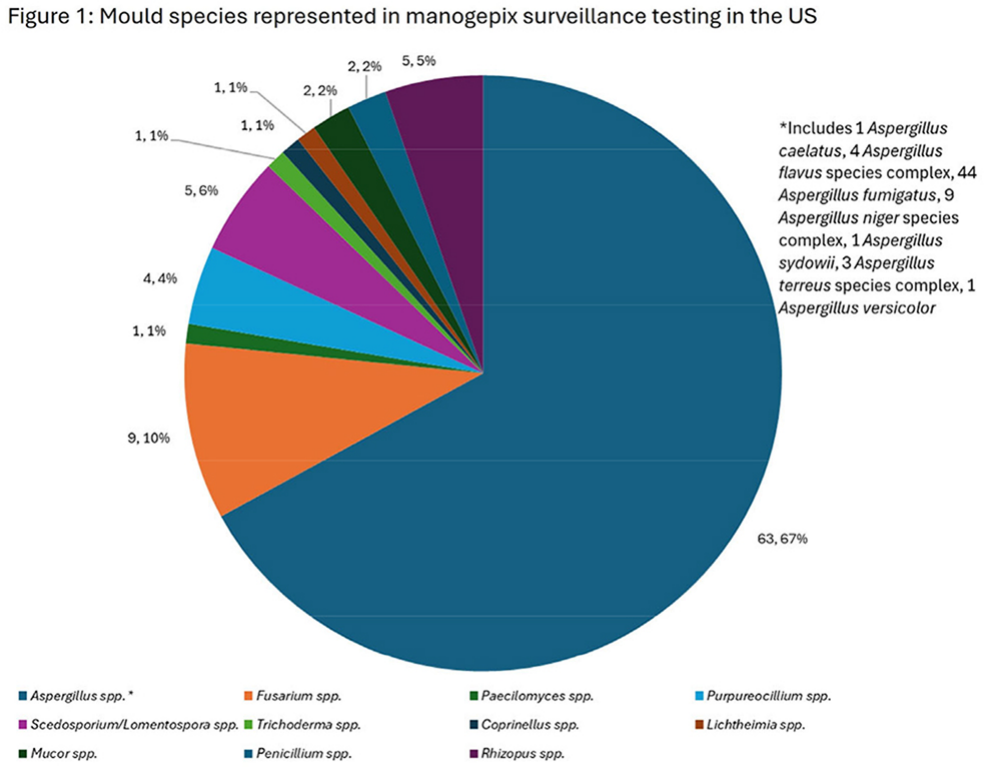

**Figure:**
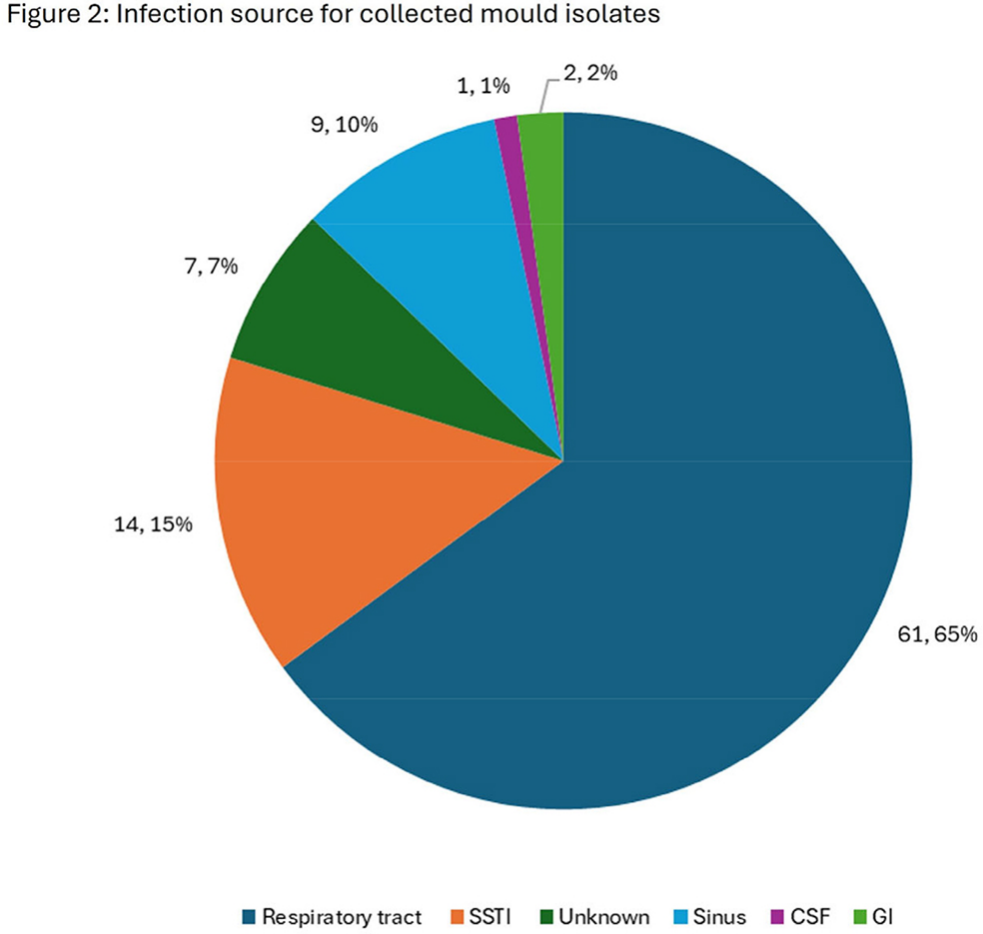

**Methods:**

A total of 94 mould isolates from 12 hospitals in 2023 were tested. Confirmatory identification was performed by MALDI-TOF or 28S sequencing. Isolates were tested by reference broth microdilution method according to CLSI guidelines against MGX, voriconazole (VRC), posaconazole (POS), isavuconazole (ISA), and amphotericin B (AMB). Susceptible, nonsusceptible (NS), wildtype (WT), or nonwildtype (NWT) isolates were identified applying CLSI breakpoints or epidemiological cutoff values (ECV). No breakpoints or ECVs are available for MGX.

**Figure:**
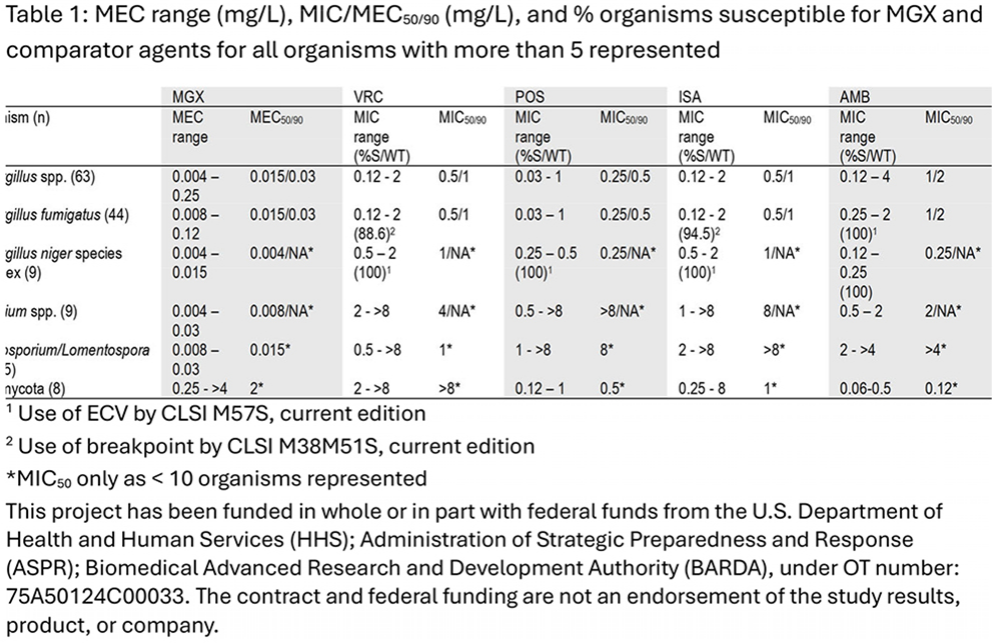

**Results:**

27 mould species and 11 genera were represented (Figure 1). Source of infection was varied (Figure 2). For 44 *Aspergillus fumigatus* (AFUM), MGX MEC_90_ was 0.03 mg/L, 16 to 32-fold lower than MIC_90_ for azoles (Table 1). 6.8% of AFUM were NS to ISA and 11.4% NS to VRC. For VRC-NS AFUM (n = 5), MGX MECs were 0.008 – 0.03 mg/L. For 9 *Aspergillus niger* species complex (ANSC), MGX MECs were 0.004-0.015 mg/L, 64 to 500-fold lower than MIC for azoles. All ANSC isolates were WT to VRC and ISA. For 9 *Fusarium* spp., MGX MECs were 0.004 – 0.03 mg/L while all azoles had limited activity (MICs ≥ 4 mg/L). For 5 *Scedosporium*/*Lomentospora*, MGX MECs were 0.008 to 0.03 mg/L, comparator agent MICs were >500-fold higher. For the 8 Zygomycota (5 *Rhizopus* spp., 2 *Mucor* spp., and 1 *Lichtheimia*), MGX MECs ranged from 0.25 to 4 mg/L, which was similar to other comparators.

**Conclusion:**

MGX has potent *in vitro* activity against mould isolates collected from invasive infections as part of a worldwide surveillance program. MGX has low MECs for challenging moulds including azole-R or azole-NWT *Aspergillus* spp. and *Fusarium* spp. and *Scedosporium/Lomentospora* spp. with elevated MICs to other agents. MGX is a promising novel antifungal agent for the treatment of invasive mould infections, including those caused by resistant isolates and warrants further development for clinical use.

**Disclosures:**

Marisa Winkler, MD, PhD, Basilea: Advisor/Consultant|Basilea: Grant/Research Support|GSK: Advisor/Consultant|GSK: Grant/Research Support|Melinta Therapeutics: Advisor/Consultant|Melinta Therapeutics: Grant/Research Support|Mundipharma: Advisor/Consultant|Mundipharma: Grant/Research Support|Pfizer: Advisor/Consultant|Pfizer: Grant/Research Support|Pulmocide: Advisor/Consultant|Pulmocide: Grant/Research Support Mariana Castanheira, PhD, Melinta Therapeutics: Advisor/Consultant|Melinta Therapeutics: Grant/Research Support

